# Be prepared for the unexpected: Mind‐stretching in ST‐elevation myocardial infarction with situs inversus totalis

**DOI:** 10.1002/ccr3.8768

**Published:** 2024-04-24

**Authors:** Giacomo Viggiani, Tareq Ibrahim, Julia Allescher, Alexander Steger

**Affiliations:** ^1^ Technical University of Munich (TUM) Munich Germany; ^2^ Klinikum Rechts der Isar, Klinik und Poliklinik für Innere Medizin I Munich Germany; ^3^ DZHK (German Centre for Cardiovascular Research), Partner Site Munich Heart Alliance Munich Germany

**Keywords:** coronary angiography, dextrocardia, percutaneous coronary intervention, situs inversus totalis, STEMI

## Abstract

Situs inversus totalis is a rare diagnosis, but the likelihood of experiencing myocardial infarction is presumed to be comparable to the general population average. In individuals exhibiting situs inversus with suspected myocardial infarction, ECG recording, including right precordial leads, is crucial for diagnostic assessment. Coronary angiography and intervention should be performed with standard equipment using inverted maneuvers and radiographic projections.

## 1 INTRODUCTION

Situs inversus totalis is a rare congenital condition in which the thoracic and abdominal organs are transposed in a mirror‐image fashion, compared to their normal anatomy (*situs solitus*). This condition presents itself with a prevalence of 1:10,000 and is more frequent in males.^1^, ^2^ The incidence of acute coronary syndromes in this condition is presumed to be similar to that in the general population.^3^ Situs inversus may be challenging for the attending cardiologist in the acute setting of a myocardial infarction, particularly if previously unknown. In patients with inverted organ laterality, the challenges are posed by the proper diagnostic recognition and by a proper interventional approach. Because of the rarity of the condition, interventional cardiologists are seldomly trained to perform coronary interventions in the setting of situs inversus.

## 2 CASE HISTORY/EXAMINATION

**FIGURE 1:**
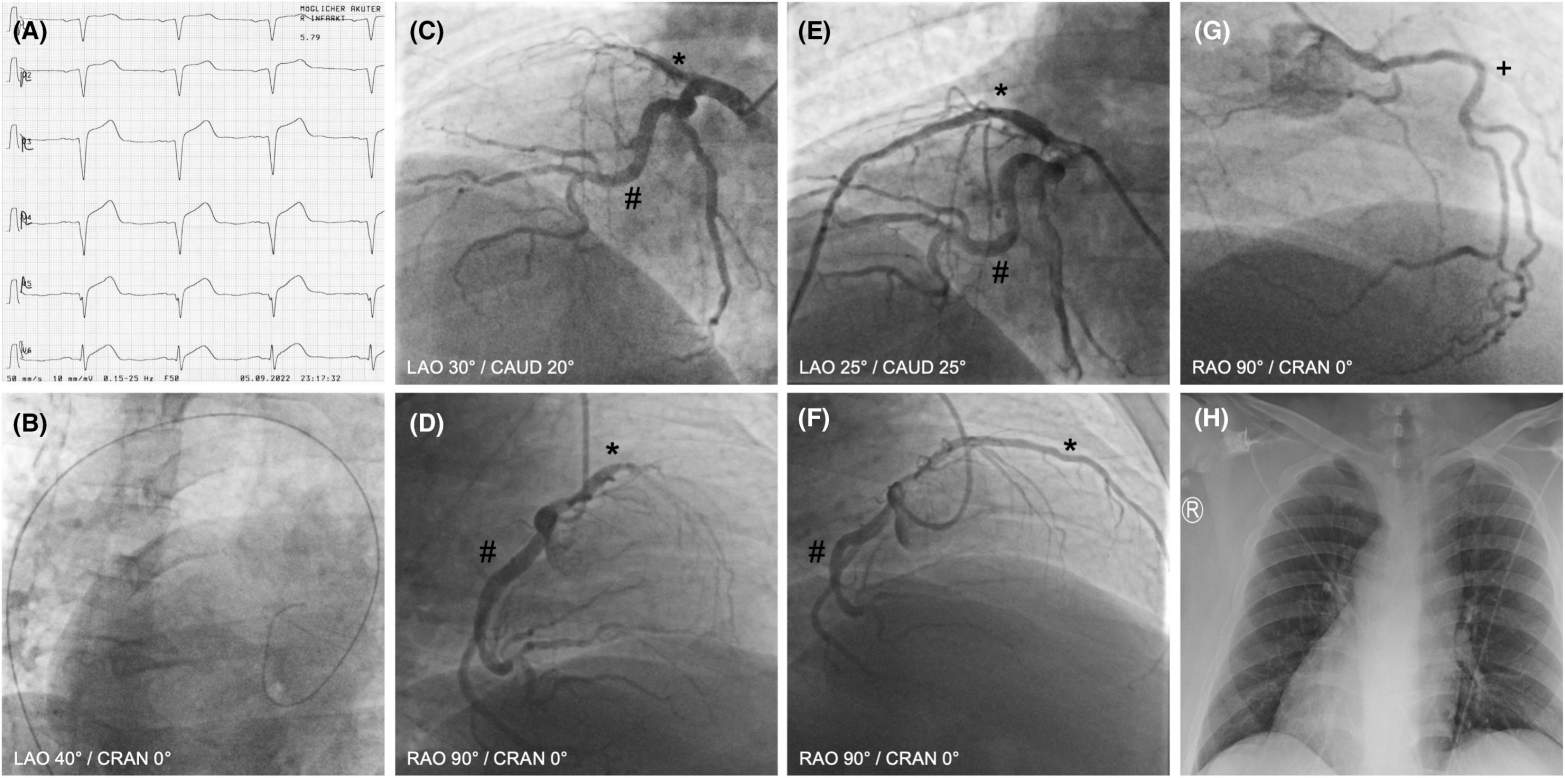
(A) Electrocardiogram showing right precordial leads with ST‐segment elevation in V_2R_ to V_5R_. (B) Positioning of the guidewire showed the aortic arch bending towards the right side, suggesting organ inversion at LAO 40°/CRAN 0°. (C) Coronary angiography showing proximal thrombotic occlusion of a dominant RIVA at LAO 30°/CAUD 20°. (D) Coronary angiography showing proximal thrombotic occlusion of a dominant RIVA at RAO 90°/CRAN 0°. (E) Angiography after PTCA/stenting at LAO 25°/CAUD 25°. (F) Angiography after PTCA/stenting at RAO 90°/CRAN 0°. (G) Coronary angiography showing left‐sided RCA at RAO 90°/RAN 0. (H) Chest X‐ray showing situs inversus with dextrocardia. *Ramus interventricularis anterior (RIVA), #ramus circumflexus (RCx), +right coronary artery (RCA).

A 55‐year‐old male was referred with suspected acute coronary syndrome due to sudden onset of chest pain. Electrocardiogram (ECG) at admission displayed ST‐segment elevation on right precordial leads (V_2R_ to V_5R_), thus suggesting right ventricular infarction (Figure 1A).

## 3 METHODS

Immediate coronary angiography was performed. Here, first positioning of the guidewire showed the aortic arch bending towards the right side, suggesting organ inversion (Figure 1B). Levocardiography performed in 30° left anterior oblique (LAO) projection revealed evidence of anterior myocardial infarction by anterior wall hypokinesia with impaired ventricular function. Selective coronary angiograms were performed with standard 6F diagnostic catheters in inverted technique, mirroring all angiographic projections and rotating the catheters during coronary intubation in the opposite direction. A Judkins R4 catheter was used for the left‐sided right coronary artery (RCA) and an internal mammary artery (IMA) catheter for a second steep ostium of the left‐sided RCA. The right‐sided left coronary artery (LCA) was imaged with a Judkins L5 catheter, showing proximal thrombotic occlusion of a dominant ramus interventricularis anterior (RIVA) (Figure 1C,D, and Figure 1G; *RIVA, #ramus circumflexus (RCx), +RCA). Recanalization and percutaneous transluminal coronary angioplasty (PTCA) were performed through an extra back‐up guiding catheter (XB4SH) and a workhorse guidewire, with immediate flow return, followed by stenting with three drug‐eluting stents resulting in complete restoration of perfusion (door‐to‐balloon time 33 min, Figure 1E,F). Further diagnostic workup including chest X‐ray (Figure 1H) and echocardiography confirmed the diagnosis of situs inversus with dextrocardia. The patient always displayed hemodynamic and respiratory stability and was transferred to the chest pain unit for further medical observation and treatment. A guideline‐directed medical therapy was initiated including dual antiplatelet therapy (ASS and Prasugrel), a statin, a cardio‐selective betablocker, an angiotensin receptor/neprilysin inhibitor, a SGLT2 inhibitor, and a mineralocorticoid receptor antagonist. He was discharged after 4 days.

## 4 CONCLUSION

Situs inversus is a rare condition, but the incidence of acute coronary syndromes is presumed to be similar to that in the general population. Extended ECG recording with additional leads, including right precordial leads, is crucial in detecting myocardial infarction in this anatomic feature. Moreover, performing coronary catheterization with standard equipment in an inverted fashion may facilitate orientation and interpretation, as well as the interventional procedure in situs inversus.

## 5 DISCUSSION

In patients with situs inversus, the diagnosis of acute coronary syndromes, particularly ST‐segment elevation myocardial infarction, can be challenging due to the inverted cardiac anatomy. ST‐segment changes can easily be missed if only a standard 12‐lead ECG is obtained. Therefore, additional ECG leads on admission play a crucial role in the diagnostic workup. Accordingly, the current ESC guidelines for the management of acute coronary syndromes recommend the use of additional ECG leads, such as right precordial and posterior leads, in cases of suspected coronary occlusion (recommendation class I B).^4^ In the present case, ST‐segment elevations were only visible in the right precordial leads.

The inverted course of the aorta and the detection of dextrocardia on the levocardiogram can cause even an experienced interventional cardiologist to break out in a sweat. However, by keeping a cool head and applying a few logical rules, these unexpected findings can be managed successfully. In the setting of dextrocardia, the coronary arteries would appear mirror‐inverted compared to standard anatomy. Therefore, in the vast number of patients, standard coronary catheters and equipment can be used to intubate the coronary arteries. It is however necessary to rotate the catheters in the opposite direction to normal and to use inverted radiographic projections. Accordingly, levocardiography can be performed at LAO 30° and coronary intubation can be achieved at right anterior oblique (RAO) 40°.

Due to its rarity, many interventional cardiologists will never encounter a patient with the condition described in this case report. However, treating such a patient is possible with the right proceedings and adjustments. In summa, the crucial points rely on recognizing the condition promptly and on knowing how to approach the diagnostic and interventional workup, in order to work effectively and avoid wasting of time in the acute setting of myocardial infarction.

## AUTHOR CONTRIBUTIONS

**Giacomo Viggiani:** Conceptualization; data curation; investigation; methodology; project administration; visualization; writing – original draft; writing – review and editing. **Tareq Ibrahim:** Supervision; writing – review and editing. **Julia Allescher:** Writing – review and editing. **Alexander Steger:** Conceptualization; data curation; formal analysis; investigation; methodology; project administration; supervision; validation; writing – review and editing.

## 6 FUNDING INFORMATION

No Funding.

## CONFLICT OF INTEREST STATEMENT

None declared.

## CONSENT

Written informed consent was obtained from the patient to publish this report in accordance with the journal's patient consent policy.

## Data Availability

The data that support the findings of this study are available upon reasonable request from the corresponding author, GV. The raw data are not publicly available due to privacy restrictions regarding clinical data.
